# Antityphoid Inoculation

**Published:** 1901-09-28

**Authors:** 


### ANTITYPHOID INOCULATION.

Some recent experiments made by Professor Wright,[1] of Netley, in regard to the changes effected by antityphoid inoculation in the bactericidal power of the blood, tend to show that several points must be borne in mind in estimating the protective effect of such inoculations in any given case, especially the degree of reaction and the time which has elapsed after inoculation before the patient has to be exposed to infection. From the experiments which he details, it appears that when the quantum of antityphoid vaccine employed produces the familiar well-marked constitutional symptoms, a decrease in the bactericidal power of the blood, and a correspondingly increased susceptibility to typhoid infection, may supervene in the period immediately subsequent to inoculation; while upon this negative phase of increased susceptibility there may be expected to succeed, probably within a period of three weeks or less, a phase of increased bactericidal power and a greater resistance to infection. But, and this is a matter of much importance, when the quantum of antityphoid vaccine employed produces very severe constitutional symptoms, a negative phase of increased susceptibility will be produced which—and the same would appear to hold true in case of a negative phase supervening upon an actual attack of typhoid fever—may never be followed up by a positive phase of increased resistance. Thus, if the inoculation is overdone, actual harm instead of good may result. Lastly, when the quantum used is reduced to the point at which marked constitutional disturbance is avoided, a positive phase of increased resistance may be expected to supervene without the intervention of any negative phase, and in many cases within 24 hours. From all this we may conclude that the employment of large doses in primary vaccination is always inadvisable, for not only might it result in failure of ultimate benefit, but it might increase the immediate risk of infection if done in the actual presence of a typhoid epidemic. Even the employment of moderate doses, such as would give rise to marked but not unduly severe constitutional symptoms, would be inadvisable in making primary inoculations in the presence of an epidemic. On the other hand, such inoculations would seem to be appropriate where an interval of several weeks is to elapse before exposure to infection and where there are likely to be difficulties in carrying out a second inoculation. In the actual presence of typhoid infection the only appropriate proceeding would seem to be the employment of small doses of vaccine, doses which only produce a slight constitutional disturbance. In fact in all other cases this would appear to be the best method—such primary inoculations being followed up, however, by second inoculations with an increased dose of the vaccine. Clearly, if one can, as Professor Wright shows we can, estimate the existing bactericidal power of the blood, it becomes a matter of considerable importance, in such a case, for example, as where a man has already had typhoid fever, to know what resisting power he possesses before submitting him to inoculation. His blood may happen already to possess such a degree of resistance as one could hardly hope to improve by inoculation; while on the other hand it sometimes happens that an attack of typhoid leaves a resistance less than the average, in which case inoculation may be very desirable where re-exposure to infection is contemplated—*i.e.*, taking for granted that inoculation has all efficacy which is claimed for it. In regard to this latter point it must be noted that these observations now published by Professor Wright go far to explain many of the apparent failures of inoculation, those for example in which exposure soon after a severe inoculation has been followed by infection.

[1] Lancet, Sept. 14.

